# Self-Injury in Autism Spectrum Disorder and Intellectual Disability: Exploring the Role of Reactivity to Pain and Sensory Input

**DOI:** 10.3390/brainsci7110140

**Published:** 2017-10-26

**Authors:** Jane Summers, Ali Shahrami, Stefanie Cali, Chantelle D’Mello, Milena Kako, Andjelka Palikucin-Reljin, Melissa Savage, Olivia Shaw, Yona Lunsky

**Affiliations:** 1 Department of Psychiatry, University of Toronto, Toronto, ON M5S 1A1, Canada; yona.lunsky@camh.ca; 2 Centre for Addiction and Mental Health, 1001 Queen Street W, Toronto, ON M6J 1H4, Canada; ali.shahrami@camh.ca (A.S.); stefanie.cali@camh.ca (S.C.); chantelle.dmello@camh.ca (C.D.); milena.kako@camh.ca (M.K.); andjelka.palikucinreljin@camh.ca (A.P.-R.); melissa.savage@camh.ca (M.S.); olivia.shaw@camh.ca (O.S.)

**Keywords:** self-injury, autism spectrum disorder, intellectual disability, pain, sensory processing abnormalities

## Abstract

This paper provides information about the prevalence and topography of self-injurious behavior in children and adults with autism spectrum disorder and intellectual disability. Dominant models regarding the etiology of self-injury in this population are reviewed, with a focus on the role of reactivity to pain and sensory input. Neuroimaging studies are presented and suggestions are offered for future research.

## 1. Introduction

Severe self-injury is a debilitating behavior that occurs in a proportion of children and adults with autism spectrum disorder (ASD) and can have a devastating impact on their physical health, developmental outcomes, and quality of life. Watching individuals harm themselves to the point of causing visible injury and not knowing the reasons why or how to stop it is both frightening and frustrating to parents and caregivers.

The purpose of this paper is to explore the role of reactivity to pain and sensory input in individuals with ASD and intellectual disability (ID) who engage in self-injurious behavior (SIB). We provide background information about the prevalence and topography of SIB in this population and outline risk factors. We are particularly interested in chronic SIB among individuals with ASD who have more severe cognitive and communication impairments as their SIB can have the most significant consequences. We present theories regarding the etiology of SIB in this population, with a focus on reactivity to pain and sensory input. Neuroimaging studies are also reviewed and suggestions are offered for future research. Finally, we provide practical recommendations for clinicians.

## 2. Diagnostic Criteria for ASD 

Autism spectrum disorder is a neurodevelopmental condition that is characterized by social communication deficits and inflexible, repetitive patterns of behavior that are present from early in life and which result in significant limitations in adaptive functioning [1]. One very important aspect of ASD concerns sensory abnormalities, which are now included in the DSM 5. In contrast to prior versions of the DSM which categorize subtypes of conditions, the DSM 5 takes a more dimensional view, recognizing that all individuals share these common features. Although a strong genetic basis for ASD has been established, the causes are in fact heterogeneous. ASD is frequently accompanied by intellectual disability.

## 3. Self-Injurious Behavior (SIB)

### 3.1. Background Information on SIB

Self-injurious behavior (SIB) often refers to actions directed toward the self that lead to physical harm, typically in the form of tissue damage [2]. Potential forms of SIB can be observed in typically developing children. For instance, infants have been known to display persistent rhythmic head banging. The behavior occurs particularly when children are tired, alone, upset, or at bedtime and may provide vestibular stimulation [3]. Toddlers and young children may hit themselves or bang their heads during a temper tantrum [4], reflecting underlying emotional states of anger and distress [5]. These behaviors may resemble SIB in form (“proto-SIB” [6]), but do not typically result in physical injury. Moreover, children tend to “outgrow” them as they develop language and emotional regulation skills.

Self-injurious behaviors, sometimes referred to as self-harm, can be present in the general population of youth and adults. Psychiatric conditions such as borderline personality disorder are associated with deliberate self-harming behavior in the absence of suicidal intent, which is known as non-suicidal self-injury or NSSI. Examples of these behaviors include skin cutting, burning, or interfering with wound healing. NSSI in these conditions may be related to impulsivity, emotional dysregulation, and inadequate coping skills, and can be seen in part as a way for an individual to bring about relief from or gain control over intense negative emotions [7]. It is also important to recognize that NSSI can occur in individuals with ASD. In a study by Maddox and colleagues [8], a history of engaging in NSSI was reported by a number of high functioning adults with ASD who were recruited to complete an on-line survey about behavioral issues. Women with ASD were more likely to have engaged in NSSI than men with ASD, suggesting that further research on gender differences is warranted.

In addition to the above populations, SIB has a high prevalence rate among individuals with severe intellectual disabilities (ID), many of whom have co-occurring autism spectrum disorder (ASD), and the impact of this behavior can have devastating consequences for these individuals and their caregivers. The next sections provide information about the topography, impact, and prevalence of SIB in this population.

### 3.2. Topography and Impact of SIB for Individuals with ASD and ID

SIB is frequent in children and adults with ASD (occurring in up to 50% of the population) and can be understood as existing on a continuum in relation to frequency and intensity, ranging from mild and infrequent to severe and chronic. The latter types of SIB involve forceful intense contact with specific body sites which has the potential to cause lasting physical damage [9]. Repetitive or stereotyped motor movements are a key component of SIB [10]. The physical impact of SIB has been demonstrated to be of significant concern for the health and well-being of the individual. For instance, Newell and colleagues [11] conducted a biomechanical analysis of head hitting in adults with severe ID, and determined that the physical impact of their SIB was equivalent to the effects of boxing punches.

Within the ASD/ID population, head banging is one of the most common forms of SIB; other forms include head hitting, biting, scratching or picking the skin, hair pulling, eye poking, vomiting/rumination, and ingestion of non-edible substances, known as pica [12]. Individuals often engage in more than one form of SIB [13]. Physical damage may be visible on the surface of the body or injuries can occur internally [14]. Consequences of SIB include infection, scarring, concussion, accidental poisoning, fractures, eye and dental injuries, bowel obstruction [15], and even premature death [16]. Interestingly, post mortem examinations conducted on the brains of two individuals with ASD and ID ages of 24 and 27 who had a long history of head banging (but died from unrelated causes) showed neuropathological changes, including neurofibrillary tangles similar to boxers who suffered chronic repetitive head injury [17,18]. Injuries caused by SIB may require medical treatment in the form of antibiotics and anti-inflammatories, suturing, skin grafts, and surgical intervention, and emergency care is sometimes necessary [19]. SIB is also associated with reduced learning and social opportunities, placement in more restrictive settings, increased levels of family stress, and exorbitant costs for care [20].

Distinctive patterns of SIB are part of the behavioral phenotype found in over one dozen genetic intellectual disability syndromes [21]. Examples include the biting of lips and fingers in Lesch-Nyhan; self-hitting in Cornelia de Lange; the removal of finger and toe nails in Smith Magenis; and skin picking in Prader Willi syndrome [22]. In addition, ASD-like characteristics such as restricted and repetitive behavior have been reported in a number of these syndromes [23]. Animal models have been developed for SIB. For instance, SIB has been induced in rodents by administering high doses of pemoline, which is a monoamine agonist that blocks the uptake of dopamine and norepinephrine [24]. Genetically altered mice have been created for human ID syndromes that include SIB as part of their phenotype. These mouse models can be useful for conducting behavioral observations and testing new therapies, with the hopes of extrapolating findings to humans. In non-human primates, the development of SIB has been linked to negative life experiences, including being raised in isolation or separation from the mother or social group at a critical stage of development. Stress and trauma may also play a role in the development of SIB, including when non-human primates are repeatedly exposed to medical procedures [25,26].

### 3.3. Prevalence of SIB 

Prevalence estimates of SIB among individuals with ID (some of whom also have a diagnosis of ASD) can vary widely, depending in part on the methodology used for ascertainment, the definition of SIB, and the characteristics of the participants being evaluated. In one of the largest studies to date, a total population approach was used to determine the point prevalence of SIB among 1023 individuals with ID ages of 16 and older who were receiving supportive services [27]. Individuals with ID and their caregivers were interviewed to establish the presence of SIB based on well-defined clinical criteria, resulting in a prevalence rate of 4.9%. An earlier total population study yielded a 4% prevalence rate for SIB among children and adults with ID and a 33% prevalence rate among those who were identified by caregivers as having more severe challenging behavior [28]. Multiple forms of SIB were common in this group, with some individuals displaying five or more topographies of SIB. Deb and colleagues [29] studied the prevalence of SIB in children and adults with ID who were randomly selected from a social service registry. Data on SIB were obtained using a rating scale that was completed during a psychiatric evaluation. The investigators were interested in SIB that was frequent (occurring more than three times per week) and/or severe, reporting that 24% of the sample met these criteria. Unfortunately, despite the fact that children with SIB are up to 13 times more likely to require significant behavioral intervention than those without frequent SIB, they are unlikely to receive this level of support [30].

Prevalence rates of SIB among individuals with ASD and ID are higher than for individuals with ID alone [31]. Some of the reasons for this finding will be explored in the section on risk factors for SIB. Baghdadli and colleagues [32] reported a 53.2% prevalence rate of SIB among 222 children with ASD ages of 2–7 who were enrolled in their longitudinal prospective study. Caregivers were asked to complete a questionnaire to indicate whether SIB was an issue and the level of severity (mild, moderate, severe) posed by the behavior. Severe SIB was reported among 14.6% children in the study, whereas mild and moderate SIB occurred among 21.5% and 17.1% of children in the study, respectively. Richards and colleagues [33] surveyed caregivers of 149 children and adults with ASD ages of 4–39 that were recruited through an autism society. A questionnaire was used to establish the presence and describe the topography of SIB. The results indicated that 50% of individuals with ASD had engaged in SIB in the previous month. The three most common topographies of SIB were self-hitting, self-biting, and self-scratching. However, the study did not yield information about the severity of SIB exhibited. Rattaz and colleagues [34] studied SIB in 152 adolescents with ASD who were part of a large cohort undergoing longitudinal follow-up. A subset of questions from a behavioral checklist were used to document the presence and severity of SIB. Overall, 35.8% of adolescents in the study exhibited SIB; 16.6% were in the less severe group and 19.2% in the more severe group. An additional finding was that parents’ functioning and quality of life were more negatively impacted for the group of adolescents with severe SIB than low or no SIB. Duerden and colleagues [35] surveyed parents of children and adolescents who were participating in a larger study on the genetics of ASD about the occurrence and severity of SIB using data that were obtained from parent report measures. The results indicated that 52.3% of children and adolescents with ASD had engaged in SIB at some point in their lives. More severe forms of SIB such as self-hitting and self-biting occurred in 34% and 26% of children in the study, respectively, and there was a trend for older children (aged 12–19) to engage more in severe forms of the behavior.

### 3.4. Persistence of SIB

The issue of the persistence of SIB is also important to consider. Cross sectional and longitudinal designs have been used to study this issue, the latter providing important information about the natural history of SIB. Dimian and colleagues [36] studied the evolution of SIB precursor behaviors in a group of infants and toddlers (*N* = 235) at high risk for a diagnosis of ASD by virtue of having an older sibling with the disorder. Children were part of an ongoing longitudinal study and were assessed at 12 and 24 months of age. Baseline information was gathered in regard to children’s cognitive and adaptive functioning, as well as their scores on a scale that measures the occurrence and severity of different types of SIB and repetitive behavior. The point prevalence of SIB was 39% at 12 months of age and 32% one year later. SIB persisted in 48% of children at the one-year follow-up. The relative risk of engaging in SIB at 24 months of age was 1.85 times higher for children who went on to meet clinical criteria for a diagnosis of ASD (20% of the total sample) versus those who did not. Rice and colleagues [37] followed the developmental course of SIB over a time span of 18 years using data from a caregiver report measure that was administered a total of five times. The participants in the study consisted of groups of children and adults with ASD, ID from heterogeneous causes, and Prader Willi syndrome, all of whom were functioning in the mild-to-moderate range of ID. Self-hitting and self-biting were more common in the ASD group than the two contrast groups and rates of head banging and skin picking among individuals with ASD increased with age. Richards and colleagues [38] conducted a three-year follow-up of 67 children (age range of 10–17) with ASD and ID from a non-clinical sample that was recruited from a parent support organization. Children’s caregivers completed questionnaires that measured challenging behavior, as well as behaviors associated with ASD and broader mental health and developmental concerns. The results indicated that self-injury persisted in 77.8% of children who exhibited the behavior three years’ earlier and hitting self with body remained a prominent concern.

### 3.5. Risk Factors for SIB 

In addition to describing the frequency, severity, and duration of SIB, it is important to identify the determinants of SIB to help guide timely assessments and interventions [39]. Risk factors include individual characteristics and aspects of the physical and social environment. A meta-analysis of 22 prevalence and case-control studies that provided information on risk markers for challenging behavior in individuals with ID revealed that the likelihood of displaying SIB was significantly increased among individuals with ASD and those with more severe intellectual and communication impairments [40]. Due to limited data, however, it was not possible to tease apart the relative contribution of each factor to SIB. Murphy and colleagues [41] studied 157 children with ASD ages of 3–14 who were receiving ABA intervention to determine the prevalence of challenging behavior among this group, as well as to identify risk factors for specific topographies of behavior. One-third of children displayed SIB along with aggression and stereotyped behavior. The more common forms of SIB included self-biting and head hitting. More severe SIB was associated with greater impairments in cognitive functioning. McTiernan and colleagues [42] used multiple regression to identify predictors for the frequency and severity of SIB in 174 children with ASD ages of 3–14 who were enrolled in ABA programs or received varied interventions. Overall, 48.9% of children in the study displayed SIB; having a lower IQ was associated with a higher severity and frequency of SIB. Duerden and colleagues [35] used hierarchical regression analysis to identify predictors of SIB in 250 children and adolescents with ASD with differing levels of cognitive abilities. Significant factors in predicting SIB also proved to be core features of ASD—i.e., sensory processing abnormalities, insistence on sameness, and social communication impairments. A lower non-verbal cognitive ability was also predictive of SIB. Dimian and colleagues [36] used logistic regression to identify child characteristics that predicted SIB at 24 months of age for infants who were at high risk for a diagnosis of ASD. The results indicated that the presence of SIB at 12 months and lower developmental skills were predictive of SIB one year later.

Richards and colleagues [38] studied the persistence of SIB after three years in a group of children with ASD. When the sample was separated into groups based on the presence or absence of SIB at follow-up, children from the persistent SIB group demonstrated lower levels of verbal communication and self-help skills, as well as greater problems with low mood, social interactions, impulsivity, and stereotyped, compulsive, and repetitive behavior. The investigators speculated that severity of ID and ASD symptomatology and problems with behavioral inhibition were important correlates of SIB. They went on to state that low mood and SIB may be related to the presence of untreated pain.

Soke and colleagues [43] used data from two large monitoring and treatment networks for children with ASD ages of 2–18 to investigate the risk factors for SIB. Significant relationships were found between SIB and low skill levels, a history of developmental regression, the presence of sensory impairments and neurological conditions, and younger age, as well as the co-occurrence of challenging behavior and sleep problems. Richards and colleagues [44] added health conditions and problems with behavioral inhibition to the list of potential correlates of SIB. Their sample consisted of 424 children and adults aged 6–61 who were receiving specialized ASD services. In line with the results of previous studies, SIB was prevalent (occurring in 45.7% of children and 49.1% of adults), with severe SIB being found in a smaller subset of children (18%) and adults (19.9%). Predictors of severe SIB in children included potentially painful digestive and skin problems along with greater impairments in functioning and impulsive and overactive behavior.

In summary, SIB is heterogeneous in nature and can have different etiologies and correlates. Some of these correlates include core features of ASD (social-communication deficits, restricted and repetitive behavior, abnormalities in sensory processing) along with a younger age, low developmental level, co-existing challenging behavior, and painful or unpleasant health problems.

## 4. Models of SIB

### 4.1. Behavioral Model

Different models have been offered to account for the emergence and persistence of SIB in individuals with ID, many of whom have a diagnosis of ASD. One of the most influential models for SIB comes from the field of applied behavior analysis. The behavioral model provides a useful framework for assessing SIB and developing intervention approaches. In a seminal paper, Carr [45] summarized a number of hypotheses regarding the motivation or “reasons” for SIB. According to Carr and others, individuals with ID learn over time to associate the occurrence of SIB with what happens afterward (“consequences”). The likelihood of SIB occurring again in the future is increased as a result of the desirable outcome that is produced following its occurrence. Some of the outcomes that increase the future probability of SIB occurring may be socially mediated, i.e., the provision of social attention or access to a preferred item or activity (positive reinforcement) or the opportunity to avoid or escape from an unpleasant or painful situation or stimulus (negative reinforcement). SIB can also be non-socially mediated, i.e., serves a self-stimulatory function and occurs in the absence of external consequences. Carr also proposed that SIB may be related to but not driven exclusively by biological or organic factors that could give rise to physical conditions or altered pain thresholds.

Iwata and colleagues [13] subsequently described a methodology to evaluate the relationship between SIB and environmental factors in a group of children and adolescents with ID from diverse etiologies. This methodology is known as functional analysis. In their study, experimental conditions were designed to resemble real-life consequences that were provided following the occurrence of SIB, consisting of statements of social concern or disapproval and briefly stopping challenging academic tasks. Individuals were also observed while alone in a room without other forms of stimulation being present as a means to determine whether SIB generated sensory (biological) reinforcement. While rates of SIB varied across individuals, a consistent pattern of responding in specific conditions emerged in some cases. This finding was viewed as evidence that SIB is associated with and can be maintained by environmental factors for some individuals and may be maintained by internal factors in others. Knowledge of these specific relationships is gained through the process known as functional analysis and has important implications for designing individualized treatment plans. These plans typically include antecedent and consequent strategies. In addition, a crucial component of most treatment plans is the focus on teaching a socially appropriate behavior that is functionally equivalent to the SIB as a means of replacing the challenging behavior. An example of this includes teaching communication skills to produce the same desired outcome to “replace” SIB [46]. Functional behavioral assessment technology, which includes functional analysis, has become a mainstay in the assessment and treatment of challenging behavior such as SIB in individuals with ID and ASD [47]. The technology has been extended to consider the impact of health problems such as allergies, otitis media, menstrual pain, and digestive and sleep problems on rates of problem behavior including SIB [48].

In addition to the consequences that maintain SIB, it is also important to identify specific details about the psychosocial and biological context in which it occurs [49]. This biological context includes a complex interplay among medical, neurochemical, and genetic factors. Neurochemical correlates of SIB are gleaned from studies in animals and humans and may involve dysregulation in the dopamine, serotonin, glutamate, GABA, and opioid systems [50]. As part of this broader biological context, alterations in processing sensory input and/or pain and discomfort are starting to gain recognition as potentially important factors with regard to SIB and will be the focus of the next sections.

### 4.2. Neurobiological Models of Pain and SIB

Over the years, different perspectives have been offered regarding the role of pain in SIB. Pain is a complex biological phenomenon that has sensory and emotional components. Pain acts as a warning system to protect us from further harm and allow injured tissues to heal. It also evokes behavior to bring about relief or comfort. Pain can differ in intensity (ranging from mild to severe), onset (sudden to gradual), and duration (lasting from a few milliseconds to months or even years). Acute pain usually occurs suddenly and can be traced to an injury or disease, whereas chronic pain persists over longer periods of time even after an injury has healed or an illness has ended and can become a problem onto itself.

Specialized sensory receptors (“nociceptors”) in the skin and internal organs carry signals along small nerve fiber pathways to the dorsal horn of the spinal cord, where they come into contact with interneurons that play an inhibitory or excitatory role. If inhibitory interneurons are blocked, secondary projection neurons transmit the nociceptive signal to areas in the brainstem and brain where they are interpreted as pain [51]. Different brain regions that are involved in cognition, emotion, sensation, and pain act together to support the experience and modulation of pain [52].

SIB is a complex behavior that likely involves alterations in circuits that connect multiple brain regions and dysregulation in various neurotransmitter systems (involving serotonin, opioids, dopamine, glutamate, and GABA) [50,53]. Sandman [54] proposed an opiate hypothesis of SIB in individuals with ASD, arguing that individuals may engage in the behavior to bring about the release of endogenous opiates (such as endorphins) which result in a “high” that can become addictive. He also postulated that individuals with ASD may have altered sensory thresholds, possibly due to high circulating levels of endorphins which rendered them less sensitive to pain. Opioid antagonists such as naltrexone may reduce levels of SIB by blocking the individual’s “high” or increasing the individual’s sensitivity to pain. Naltrexone has been helpful for reducing the frequency of SIB in some individuals with ID and ASD [55].

Symons [9] proposed a link between behavioral and biological models of chronic SIB. Factors such as where SIB occurs on the body and the intensity of SIB may have a bearing on the sensation of pain or noxious stimuli. For instance, hits to the head and face may be reduced when naltrexone is administered but not when SIB is directed toward other areas of the body. Varying intensities of SIB may be regulated by different sensory pathways and neurochemicals. Symons also presented evidence of abnormal gaps between epidermal nerve fibers (ENF) in individuals who engage in chronic SIB, which could alter or disrupt pain sensitivity and the resultant aversive qualities of SIB. Peebles and Price [56] raised the possibility that pain thresholds may be normal in some individuals with ID and SIB, but the amplification of pain signals may be diminished so that pain doesn’t act as a deterrent for SIB or a more intense pain stimulus is required to activate anti-nociceptive processes via descending inhibitory neurons.

## 5. Role of Pain in SIB

### 5.1. Measuring Pain in Individuals with ASD 

In verbal individuals with ASD, self-reporting about painful events is possible although sometimes difficult. For instance, Ely and colleagues [57] interviewed 40 children with ASD ages of 6–17 soon after they had undergone a surgical procedure to obtain first-hand accounts about their pain experiences. Children were also provided with visual tools to augment their verbal reports by helping them to identify the area of their body that hurt and quantify the intensity of their pain. In terms of strategies used to help to ameliorate their pain, children mentioned distraction, relaxation, taking medication, and seeking out a parent. Other methods that have been used to gather information about children’s responses to pain include caregiver questionnaires and rating scales, structured behavioral observations, and physiological measures. Rattaz and colleagues [58] evaluated the reactions of 35 children with ASD ages of 4–6 who were undergoing venipuncture for routine purposes, comparing them to children with ID and typically developing children matched for the developmental level. Children’s reactions to the painful procedure were measured using the heart rate and coding of facial actions and scores on the Noncommunicating Children’s Pain Checklist—Revised (NCCPC-R [59]), which was designed to be used by observers to document the presence and intensity of pain responses in children with cognitive impairments. While all three groups showed a similar pattern of responding to the procedure, the behavioral reactions of children with ASD were more prolonged and could be viewed as evidence that other elements of the situation were distressing to them. The venipuncture procedure provides only one type of opportunity to observe individuals’ responses during routine medical procedures and could be expanded to include other experiences, such as dental treatments [60].

Courtemanche and Black [61] evaluated behaviors indicative of pain in a sample of 51 children who were referred for an evaluation of ASD and developmental concerns. Children’s parents were instructed to complete pain ratings on the NCCPC-R in consideration of their behavior during the previous 2-h and 1-week time periods. Children’s classification into ASD versus non-ASD groups did not differentially impact their total scores on the NCCPC-R for both time periods. Interestingly, children with more severe symptoms of ASD received lower scores on the pain measure, raising the possibility they may express pain in different ways than children with less severe symptomatology.

### 5.2. Pain Experience and Expression in Individuals with ASD

At one time, individuals with ASD who engaged in SIB were viewed as having a high tolerance for pain, in part due to their hypo-responsiveness or lack of reaction to a range of positive and negative stimuli, as well as the fact that pain should ordinarily act as a deterrent for self-harm [62]. An unfortunate outcome of this belief is that pain assessments may not be carried out routinely for individuals with ID, including those with ASD [63]. Allely [64] conducted a systematic review of research on pain responses in individuals with ASD, finding several case reports that provided some support for pain insensitivity. However, evidence from experimental studies challenged this view and instead suggested that individuals with ASD experience pain but may express it in a different or unusual manner. Moore [60] examined studies that provided information about the experience of pain and pain expression in individuals with ASD. A review of studies based on clinical observations and experimental investigations using standardized pain stimuli (e.g., electrical or thermal stimulation) failed to yield evidence of hypo-sensitivity to pain or increased pain thresholds in individuals with ASD, but did raise the possibility that they may display different social communicative behaviors during pain episodes. For instance, children with ASD may express negative emotional reactions such as screaming or noncompliance when experiencing pain which could mislead caregivers into looking for non-pain related explanations for their behavior [65].

Tordjman and colleagues [66] measured the pain reactivity of 73 children and adolescents (mean age of 11.7 years) with ASD and ID (72% of whom were considered to be nonverbal). A control group consisted of 115 children without ASD who were matched on the basis of age, sex, and stage of pubertal development. A behavioral scale was used by parents to categorize their child’s reaction to pain in response to life events such as illness or an accident. The five different categories of pain reactivity were: paradoxical, absent, hyporeactive, normal, and hyperreactive. The same scale was used by medical staff to categorize children’s response to pain during a venipuncture procedure. The presence of challenging behavior (including SIB) was also documented during the procedure. Children’s heart rate was monitored before and after the procedure and their plasma β-endorphin concentrations were measured. Over half (55%) of children with ASD were classified by medical staff as showing absent or hypo-reactive responses to pain during venipuncture versus 39% of children in the control group; normal or heightened reactions to pain were identified for 38% of children with ASD versus 60% of controls. Moreover, a proportion of children with ASD engaged in SIB immediately after the procedure versus none of the controls. Heart rates were higher for children in the ASD group than the control group before, during, and after the blood drawing procedure. β-endorphin plasma levels were higher in the ASD group than controls in general, and were higher in children with more severe ASD than children with less severe ASD and controls. The investigators suggested that this finding may be reflective of increased stress rather than evidence of opioid functioning. Taken together, the findings were viewed as evidence that individuals with ASD may “feel” pain but may not always “show” pain in the same way as others.

### 5.3. Link between Pain and SIB

As mentioned previously, verbal individuals with ASD are better able to articulate the source of their pain and discomfort than nonverbal individuals for whom the presence of pain must be inferred through observation of behavioral indicators. Symons and colleagues [62] investigated a possible link between pain and SIB by recruiting a group of 35 adults with ID ages of 29–51 and chronic and severe SIB that required ongoing behavioral and/or medical management. A non-SIB control group was matched on the basis of age, gender, level of functioning, and medication usage for behavioral disorders. Both groups lived in specialized residential settings and did not have identifiable pain-related conditions. For each individual, caregivers were asked to rate the presence of pain indicators in the previous week using a modified version of the NCCPC. Individuals from the SIB group received a higher total score on the NCCPC than the non-SIB controls, establishing a link between SIB and pain indicators. It would be of interest to investigate whether the SIB and non-SIB group differed with respect to other variables that influence how pain is experienced and expressed, such as the presence of sensory abnormalities or stereotypic behavior. There is also the ongoing question of whether engaging in SIB produces pain or occurs in response to pain, as well as the impact of having chronic versus episodic SIB.

Symons and Danov [67] studied pain responses prospectively in a six-year-old boy who developed SIB (lip and tongue biting, eye poking) following brain surgery to remove a tumor. The boy’s pain responses were measured several times per day via maternal ratings using the NCCPC-R, while the Self-Injury Trauma Scale [14] was used to document the frequency of his SIB. Pain ratings were significantly higher during time periods when the child engaged in SIB. Due to the correlational nature of the study, it was not possible to tease apart whether the child was engaging in SIB because of pain or if pain was a by-product of SIB that was related to nerve dysfunction following brain surgery that altered his sensory experiences.

In another study, a 13-year-old boy with ASD and life-threatening SIB had deep brain stimulation (DBS) electrodes implanted in his basolateral amydala [68]. The boy had a long-standing history of SIB that had worsened to the point that he was required to be restrained almost continuously. He also displayed sleep disturbance and signs of anxiety and had been treated with a variety of psychotropic medications that did not produce long-lasting benefits. Prior to and following brain surgery, the intensity of the boy’s SIB and his symptoms of ASD were tracked. Several months after activation of the DBS, the boy did not require restraint to control his self-injury but it was noted that short-term SIB could sometimes be triggered by physical illness or unexpected changes in his environment. Neurostimulation was also associated with a reduction in anxiety, irritability, and stereotyped behavior and improvements in the boy’s sleep and his ability to modulate his mood. This is perhaps an extreme example but highlights the complex array of factors that can be associated with SIB.

Courtemanche and colleagues [69] studied the relationship between behaviors used to express pain and challenging behavior (SIB, aggression and stereotypies) among 51 children under the age of seven who were referred for an evaluation of ASD and developmental concerns. Children were placed into one of three groups (no SIB; infrequent and/or mild SIB; daily and moderate/severe SIB) on the basis of their scores on an SIB subscale from a behavioral questionnaire. Pain-related behavior was assessed by asking parents to complete the NCCPC-R for the preceding 2 h and 1-week time periods. Children displaying more frequent and severe SIB received significantly higher one-week total scores on the pain measure than children without SIB. While there was a relationship between SIB (in addition to aggression and stereotypies) and pain behaviors, SIB was not related to a diagnosis of ASD or children’s developmental level. The investigators speculated that challenging behavior as detected by the pain scale may be an expression of children’s agitation or distress during daily events.

Goldschmidt [70] described the case of “Paul”, a 20-year-old nonverbal adult with ASD who developed SIB after falling and suffering an injury to his leg and ankle. Paul was reportedly oblivious to his injury initially (failing to show a response to acute pain) until it resulted in changes in his routine. Behavioral changes that were noted in the months following his injury were viewed as an emotional reaction to the physical and emotional trauma he had suffered and included the emergence of obsessive compulsive symptoms, self-injury, loss of appetite, and sleep problems. Neuropsychiatric perspectives have been offered on SIB in individuals with ASD, with the view that SIB could be related to unpleasant symptoms associated with anxiety, panic, or depression and that treatment of an underlying mental health issue with psychotropic medication may bring about a reduction in SIB [71]. This situation is easier to sort out when individuals are capable of verbal reporting. For nonverbal individuals, the outward expression of emotions (through facial actions, vocalizations, motor movements) may provide a clue about their private internal states but are not always easy for observers to recognize and interpret or to identify the underlying cause. An understanding of the temporality of events can be helpful for sorting out whether challenging behavior occurs in response to negative emotions or could be linked to another factor such as pain or physical discomfort [72].

## 6. Sensory Abnormalities in ASD

Along with impairments in communication and social behavior, abnormal responding to sensory input is extremely common in individuals with ASD [73] and is considered a core feature of the disorder [1]. Problems modulating sensory input occur across multiple modalities and can have a significant impact on the lives of individuals with ASD. Sensory abnormalities have been linked to challenging behavior, mood problems, and impaired adaptive skills [74]. Individuals with sensory abnormalities can display heightened negative reactions (hyper or over-responsiveness) or decreased or slower reactions (hypo or under-responsiveness) to particular stimuli. Behavioral descriptions of these phenomena range from extreme reactivity and avoidance to apparent obliviousness or indifference. For instance, many parents are initially concerned that their children with ASD are deaf due to their lack of response to speech. However, these same children may display extreme sensitivity to environmental noises such as a vacuum or blender. It is important to highlight that an individual can show both hyper- and hypo-responsiveness to a range of stimuli. Sensory seeking behavior is a third element of sensory processing challenges and is manifested by a strong interest in or preoccupation with particular sensory experiences [75].

## 7. Link between Sensory Abnormalities, Pain or Discomfort and SIB

Much of what we know about sensory abnormalities comes from the accounts of more able individuals with ASD. Kirby and colleagues [76] interviewed 12 children aged 4–13 with high functioning ASD to gain first-hand information about their sensory experiences. Some children spoke of having vivid physical reactions to sensory input to the point where they felt pain and discomfort and reported feeling better once the experience had ended. They also described fear and anxiety in anticipation of subsequent re-exposure to these stimuli. Elwin and colleagues [77] performed a content analysis of the autobiographies of ten adults with confirmed diagnoses of high functioning autism or Asperger syndrome. The focus of interest was the authors’ descriptions of their reactions to a range of sensory stimuli. Hypersensitivity in the form of extreme positive or negative reactions to external stimulation was reported by all participants. Negative reactions reflected problems modulating sensory input, such that bright lights could induce a feeling of physical sickness, the sound of a fog horn could be perceived as excruciating, and the noise of children talking could be described as tormenting. Hyposensitivity was also reported by nine of ten authors, most commonly in reference to pain or internal sensations. Interestingly, fluctuations in sensitivity (shifting between hyper-responsiveness and hypo-responsiveness) could occur within the same individual.

Sensory abnormalities also exist in lower functioning individuals who have greater difficulty articulating the source of their discomfort and often lack socially appropriate skills to cope with it. These problems could play a role in the development and maintenance of challenging behavior such as SIB. Gonthier and colleagues [78] asked caregivers of 148 adults with ASD ages of 19–59 with severe-to-profound ID who lived in specialized care facilities to complete sensory profiles and behavioral rating scales. Using hierarchical cluster analysis, individuals were placed in different groups based on their pattern of sensory abnormalities. The researchers found a link between hypo-responsiveness to stimuli and SIB, speculating that SIB was used to provide self-stimulation. Associated problems found in this cluster of individuals included difficulties with social relationships, aggression, an irritable mood, and evidence of anxiety and other emotional disorders.

In the case of hyper-responsiveness, sensory input may be perceived as being highly unpleasant or even painful by many individuals with ASD. Kern and colleagues [79] refer to sensory overload or sensory defensiveness which is manifested as avoidance of discomfort associated with problematic sensory experiences. SIB could serve in some cases as a communication that sensory experiences may be intolerable.

## 8. Neurological Correlates of Pain and Sensory Processing in ASD

Neuroimaging techniques provide a non-invasive way to study regions of the brain that are involved in the perception and evaluation of chronic pain. These techniques include positron emission tomography (PET), electroencephalogram (EEG), motionless electromagnetic generator (MEG), single photon emission computed tomography (SPECT), and magnetic resonance imaging (MRI) and have been used to study individuals with a variety of chronic pain-related conditions such as arthritis, back pain, fibromyalgia, and recurrent migraines [80]. Evidence shows altered functional and structural changes in brain regions or networks in response to chronic pain and that sensory, motor, cognitive, motivational, and emotional processes can be impacted.

Failla and colleagues [81] used functional magnetic resonance imaging (fMRI) to measure the neural response to sustained thermal pain stimuli (looking at the neural pain signature, which is a pattern of activity across different regions of the brain) in a group of 15 high functioning adults with ASD and 16 non-ASD controls who were matched for age, gender, and IQ. Individuals had a heat stimulator applied to their lateral calf while they were in an MRI scanner. The temperature of the stimulator was raised gradually until they indicated feeling heat pain. fMRI results indicated that both groups showed a similar initial neural response to the heat induced pain; however, adults with ASD showed a reduced response to prolonged painful stimulation compared to controls, a finding the investigators speculated may reflect differences in strategies for coping with pain.

Duerden and colleagues [82] studied the relationship between SIB and cortical development in 30 children and adolescents with ASD (aged 7–15, mean IQ in the average range) who were recruited through an autism research unit. A control group of typically developing children was matched for age. Parents of children with ASD completed a standardized questionnaire on repetitive behavior that required them to indicate whether different forms of SIB had occurred in the previous month and the extent of the problem posed by the behavior (mild, moderate, or severe). They were also questioned about the location, frequency, and duration of SIB and were asked to rate their child’s reactivity to pain compared to children without ASD. Children were divided into groups with a high incidence of SIB and low or no SIB. The duration of children’s SIB ranged from six months to over eight years and several children were reported to engage in SIB daily. Children underwent MRI and diffusion tensor imaging (DTI). The results were reflective of structural brain changes; higher self-injury scores were associated with reduced grey matter in somatosensory brain areas and possible disruptions in thalamocortical white matter fiber pathways. The investigators speculated that these alterations could reflect disruptions in brain development or changes brought about by engaging in SIB.

Nonverbal individuals with ASD are often excluded from the type of investigations summarized previously due to cognitive and communication difficulties which impact their ability to cooperate with procedures; however, their data are very important for measuring pain responses and mechanisms. Several studies demonstrate that it is possible to teach individuals with ASD and ID to cooperate with medical and neuroimaging procedures. Nordahl and colleagues [83] trained 17 children with ASD ages of 9–13, many of whom had co-morbid ID, to lie still in an MRI scanner for 5–10 min. Children were not excluded from the study if they had SIB. A task analysis of the steps involved in undergoing an MRI scan formed the basis of a training protocol. Initial sessions took place in a mock-up that was built to resemble an actual scanner and provide realistic sensory experiences. A variety of behavioral strategies were used during step-by-step teaching sessions, including modeling, offering choices, priming, and providing visual feedback. Once children demonstrated success in the mock scanner (achieved in two teaching sessions or less), they underwent a real MRI procedure. While the number of scans needed to capture high quality MRI images varied among children, eventually all were successful. Grider and colleagues [84] presented details of an approach used to teach a 21-year-old male with ASD who lived in a group home to cooperate with having his blood drawn. A 12-step task analysis of the venipuncture procedure was created and followed. Sessions took place in the nurse’s office with the nurse performing the medical procedure. Key elements of the intervention included graduated exposure to materials and procedures used for the blood draw, distraction with a favorite video, and praise for compliance. Following completion of training, the individual was able to tolerate having his blood drawn one and three months later. Using similar behavioral procedures, a 16-year-old adolescent with ASD and ID was successfully taught to comply with different elements of a physical exam that included listening to her heart and checking her blood pressure and abdomen [85].

## 9. Conclusions 

In this paper, we provide information about SIB in individuals with ASD and ID, particularly those with more severe cognitive and communication impairments, with a focus on the role of reactivity to pain and sensory input. Different sources for pain and discomfort exist in individuals with ASD and ID, including physical and medical causes, emotional and neuropsychiatric disorders, and sensory sensitivities. A better understanding of how these factors individually or collectively contribute to the development and maintenance of SIB are topics for further research. Continued refinement of tools and methods for measuring pain and a range of emotional responses in this population are recommended, including physiological indicators and neuroimaging techniques. Research can help inform how to organize service systems to identify those at high risk of developing chronic SIB and discover ways to provide early and sustained interventions with the goal of minimizing suffering and maximizing the quality of life of these individuals.

## 10. Practical Recommendations for Clinicians and Caregivers

- Identify those at the greatest risk for persistent SIB and intervene as early as possible;
- Consider physical and environmental conditions that can give rise to pain, chronic stress, and discomfort;
- Treat associated problems such as sleep and an irritable mood as they may impact individuals’ ability to cope with pain;
- Use tools and methods that are validated for the population;
- Teach communication and coping skills to individuals with ASD to “replace” SIB with functionally equivalent behavior;
- Provide education to caregivers about possible differences in social communication (e.g., seeking out comfort less often) to avoid an underestimation and undertreatment of pain and discomfort; also provide training on the individual’s specific pain cues;
- Teach individuals with ASD how to cooperate with medical and diagnostic procedures in order to identify and substantiate pain or stress/discomfort.
